# Translational MEMS Platform for Planar Optical Switching Fabrics

**DOI:** 10.3390/mi10070435

**Published:** 2019-06-30

**Authors:** Suraj Sharma, Niharika Kohli, Jonathan Brière, Michaël Ménard, Frederic Nabki

**Affiliations:** 1 Department of Electrical Engineering, Ecole de Technologie Supérieure, Montréal, QC H3C 1K3, Canada; 2 AEPONYX Inc., Montréal, QC H3C 4J9, Canada; 3 Department of Computer Science, Université du Québec à Montreal, Montréal, QC H2X 3Y7, Canada

**Keywords:** microelectromechanical systems (MEMS), electrostatic actuator, parallel plate actuation, optical switch, silicon-on-insulator (SOI), micro-platform, optical waveguide, silicon nitride photonics, integrated optics

## Abstract

While 3-D microelectromechanical systems (MEMS) allow switching between a large number of ports in optical telecommunication networks, the development of such systems often suffers from design, fabrication and packaging constraints due to the complex structures, the wafer bonding processes involved, and the tight alignment tolerances between different components. In this work, we present a 2-D translational MEMS platform capable of highly efficient planar optical switching through integration with silicon nitride (SiN) based optical waveguides. The discrete lateral displacement provided by simple parallel plate actuators on opposite sides of the central platform enables switching between different input and output waveguides. The proposed structure can displace the central platform by 3.37 µm in two directions at an actuation voltage of 65 V. Additionally, the parallel plate actuator designed for closing completely the 4.26 µm air gap between the fixed and moving waveguides operates at just 50 V. Eigenmode expansion analysis shows over 99% butt-coupling efficiency the between the SiN waveguides when the gap is closed. Also, 2.5 finite-difference time-domain analysis demonstrates zero cross talk between two parallel SiN waveguides across the length of the platform for a 3.5 µm separation between adjacent waveguides enabling multiple waveguide configuration onto the platform. Different MEMS designs were simulated using static structural analysis in ANSYS. These designs were fabricated with a custom process by AEPONYX Inc. (Montreal, QC, Canada) and through the PiezoMUMPs process of MEMSCAP (Durham, NC, USA).

## 1. Introduction

Over the years, micro devices with optical and microelectromechanical systems (MEMS) components known as micro-opto-electro-mechanical systems (MOEMS) have been developed for use in digital micro mirror displays [1] and laser scanners [2]. Development of such optical MEMS devices subsided due to immaturity of the technology and market penetration challenges [3]. However, with the world moving towards higher bandwidth optical fiber-based communication, MOEMS can help meet the ever-growing demand for power and transmission efficient integrated silicon photonics solutions. Conventional electronic data centers are often associated with high cost, and high energy and space consumption [4]. These technological, environmental and monetary constraints have paved the way for MEMS integration towards the development of hybrid optical data center designs such as Helios [5] and novel Scaled Out Optically Switched Network Architecture [6]. MEMS integration into data centers can reduce power consumption from 12.5 Watts per port for electronic switches to just 0.24 Watts per port for optical switches but with a re-configurability that is restricted to a few milliseconds [4]. Such data centers often rely upon 3-D MEMS with out-of-plane rotating micro-mirrors for beam steering inside an optical cross connect switch [7] using piezoelectric actuation [8] or electrostatic actuation [9,10]. Although 3-D MEMS allow the implementation of optical switches with a large number of ports, the development of such systems often suffers from fabrication and packaging constraints due to the complex structures and wafer bonding processes involved [11,12]. Thus, paving the way for simpler more affordable 2-D MEMS based integrated photonics solutions for switching applications.

Piezoelectric [13], electrothermal [14] and electrostatic [15] actuators provide precise mechanical motion at the micron scale. Piezoelectric actuators, although fast and suitable for applications with resonators, involve the use of complex piezoelectric materials such as AlN and PZT for actuation [16,17]. These materials can be integrated with silicon-on-insulator (SOI) technology to make optical switches [18] but the use of lead in PZT raises environmental concerns [19]. Alternative piezo materials, such as AlN, are difficult to reproduce with the same piezo properties because of their dependency on the film texture and dipole orientation, and that they can have large residual stress with even a slight change in the deposition parameters [20,21]. Electrothermal actuators produce large displacements at low actuation voltages but are slow, consume high power and produce heat during actuation [22,23]. This makes them undesirable for the green optical data centers envisioned for the future. Thus, low voltage electrostatic actuators based upon widely used comb drives and parallel plate designs become the right choice for planar optical switching applications [24,25]. These can also be fabricated with ease through commercial SOI microfabrication processes to validate MEMS designs before integration with optical waveguides [26,27].

Electrostatic actuators connected to a central platform have been demonstrated in the past for 2-D and 3-D MEMS based solutions such as optical scanners and cold atom detectors [28,29]. These designs have largely relied upon out-of-plane rotational motion of the central platform due to torsional beams [30,31,32]. A few translational MEMS structures exist, but they are largely designed for out-of-plane optics applications [33,34,35]. The MEMS for planar switching applications reported in the literature rely upon bringing movable waveguides closer to fixed waveguides in ON/OFF state [36,37] or as a 1 × 3 optical switch [38]. Through complex MEMS integration of soft polymer waveguides, a 2 × 2 optical switch has also been demonstrated [39]. Recent developments include planar switching done by adiabatic coupling between waveguides through vertical actuation at very low voltage [40,41], and butt coupling through in-plane rotational actuation [42].

Accordingly, in this work, we present a translational MEMS platform capable of motion along two axes using multiple electrostatic actuators. A detailed overview of a translational MEMS platform compatible with different planar optical switching configurations is presented in Section 2 along with optical design considerations. Results of EigenMode Expansion (EME) and Finite Difference Time Domain (FDTD) simulations for the butt coupling of SiN waveguides and the cross-talk between parallel SiN waveguides are also presented in this section. Alignment tolerance simulations show the potential for efficient optical switching with the proposed MEMS platform. The evolution of the actuator and spring designs along with the critical design choices made for a simple switching approach are also discussed. Design of the translational MEMS platform and the critical design parameters and dimensions are presented in Section 3. The fabrication process used, and the analysis of the fabricated devices are discussed in Section 4. In this section, the test setup used for the actuation experiments and the results obtained are also reported. A discussion of these results is presented in Section 5 and is followed by the envisioned future work and concluding remarks in Section 6. 

## 2. Design Considerations 

### 2.1. Translational MEMS Platform for Optical Switching

Previous devices developed by our research group relied upon a rotational MEMS platform for planar optical switching using SiN waveguides surrounded by a SiO_2_ cladding [42]. The device uses 5° of its total 9.5° of rotation on each side to form a crossbar switch requiring 113 V to actuate and having a 1.3 mm by 1 mm footprint. Also, the air gap closing actuator designed operates at 118 V with a minimal gap of 250 nm upon actuation. 

**Figure 1:**
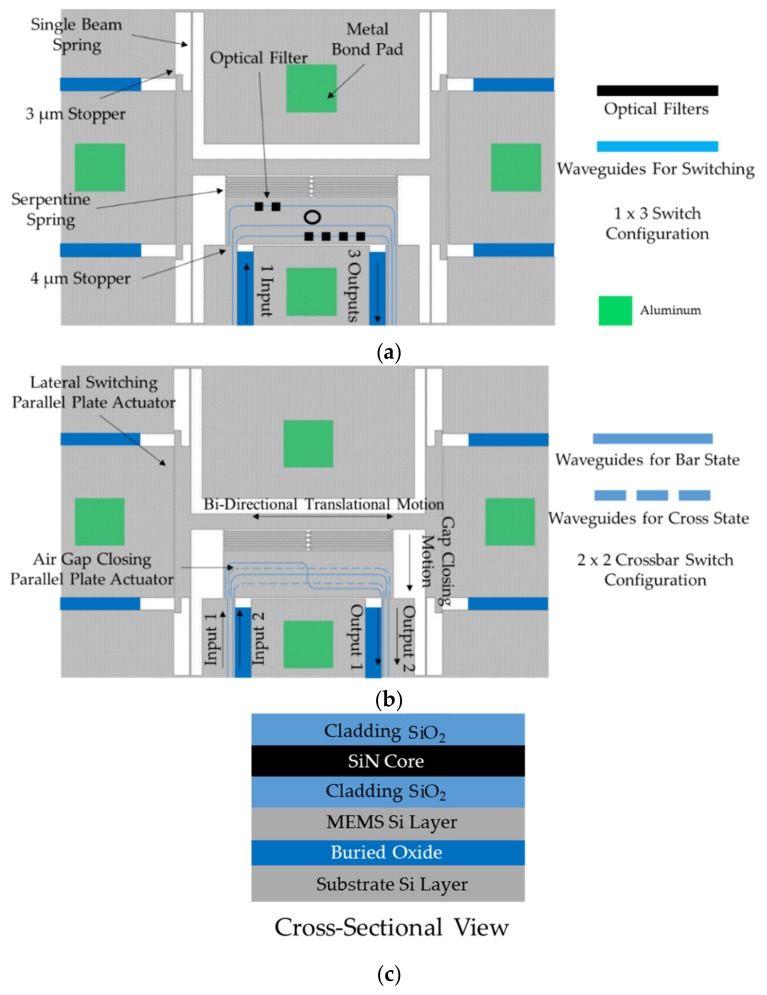
Illustrations of the proposed translational actuator: (**a**) 1 × 3 switch configuration with integrated optical filters; (**b**) 2 × 2 crossbar switch configuration; (**c**) cross sectional view of the optical MEMS stack proposed. The color scheme to represent the different materials is consistent throughout the figure.

In this work, a unique translational MEMS platform capable of bi-axial motion is proposed and demonstrated. The lateral motion of the platform is bi-directional and can provide optical switching in 1 × 3 or 2 × 2 crossbar switch configurations. The longitudinal motion of the central platform is unidirectional and designed to completely close the air gap between waveguides on the substrate and the platform to achieve highly efficient butt-coupling. The design operates at a reduced actuation voltage for both switching and gap closing motions compared to that reported in [42], and the device footprint is smaller. Figure 1 shows illustrations for the translational MEMS platform as a 1 × 3 optical switch (Figure 1a) and as a 2 × 2 crossbar optical switch (Figure 1b). These structures are meant to include integrated SiN waveguides which are not the focus on this work but that have been demonstrated in [42,43]. A cross sectional representation of the entire optical MEMS stack envisioned is also shown in Figure 1c.

In the 1 × 3 optical switch configuration, the central platform accommodates three separate SiN waveguides. The input side on the left of the platform has one waveguide on the fixed substrate. The output side on the right of the platform is designed to have three separate waveguides. Four symmetrical single silicon beam springs support the entire MEMS structure. These beams provide the necessary spring action to allow lateral displacement through parallel plate actuators on the opposite side of the platform. The central platform is connected to the lateral actuators through a central beam and a serpentine spring structure. This design choice decouples the lateral and longitudinal motion of the platform. The serpentine spring enables longitudinal displacement through a parallel plate actuator at the bottom of the platform. Whereas the parallel plate actuators on the opposite sides of the platform are designed to provide discrete lateral displacement of 3 µm on each side, the bottom parallel plate actuator is designed to close the 4 µm air gap between the platform and the substrate. The discrete lateral displacement of 3 µm on either side along with the neutral position of zero lateral displacement provides 3 switching possibilities to form a 1 × 3 switch. The platform is large enough to integrate optical filters, such as Bragg gratings and ring resonators [44,45], and it can be used to select among a bank of filters to implement discretely tunable devices. Examples of optical filters are also illustrated in Figure 1a.

Figure 1b shows the MEMS platform envisioned as a 2 × 2 crossbar switch. There are two input and output waveguides on the substrate. The actuation mechanism and operational parameters remain the same as the 1 × 3 switch configuration, but the platform accommodates four SiN waveguides. When the platform is actuated to the right, the waveguides on the platform shown as solid lines in Figure 1b are aligned with the input and output waveguides. In this position light travels from input 1 to output 1 and from input 2 to output 2, creating the ‘bar’ configuration. When the platform is actuated to the left, waveguides on the platform shown as dotted lines in Figure 1b are aligned to the input and output waveguides. The optical signal then propagates from input 1 to output 2 and from input 2 to output 1, creating the ‘cross’ configuration. In both configurations, the gap closing mechanism provides highly efficient butt-coupling between the waveguides. The discrete motion of the platform also eliminates optical losses due to displacement / voltage fluctuations in the system, as the MEMS platform is designed to operate in the pull-in state for both the lateral and longitudinal actuators.

### 2.2. Optical Design Considerations

Our work involves the validation of the MEMS [27] structures, prior to employing the commercial process enabling the addition of optical waveguides which has been developed by our group in collaboration with AEPONYX. The commercial process used to validate the MEMS requires a minimal gap of ~3 µm. This constraint leads to a significant air gap between the fixed and moving waveguides envisioned in a planar optical MEMS device. In the previous rotational MEMS developed by our group, the input and output waveguides were located on top of the gap closing actuator due to design constraints [42]. This enables the air gap to be reduced to only 250 nm as the rotational platform that is grounded cannot come in contact with the gap closing actuator that is kept at a high DC voltage. If the two come in contact, shorting during actuation would damage the MEMS device. This phenomenon can be prevented by dimpled structures but leads to a residual air gap even after gap closing. However, on the translational MEMS platform shown in Figure 1, the input and output waveguides are separated from the gap closing actuator. The air gap between the platform and the fixed section of the switch (with input / output waveguides) is designed to be 4 µm whereas the gap for the bottom parallel plate actuator is designed to be 6 µm. As a result, the platform and the fixed section of the switch can both be grounded to eliminate shorting during gap closing actuation. This provides complete gap closing between waveguides eliminating any significant residual air gap. 

**Figure 2:**
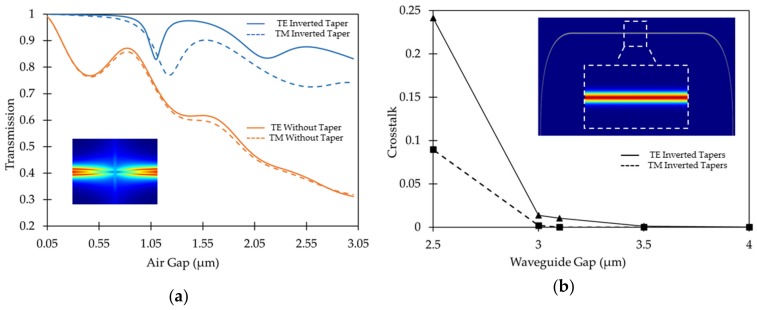
Optical simulation results for: (**a**) EME analysis showing transmission efficiency for TE and TM modes between two butt-coupled SiN waveguides as a function of air gap with and without inverted tapers. The inset shows the top-view of the magnitude of the electric field of TE mode for butt-coupling with inverted tapers at a gap of 500 nm; (**b**) 2.5D FDTD analysis showing cross-talk for TE and TM modes as a function of the gap between two SiN waveguides with 90° bends and 75 µm bending radius. Inset shows top-view of the magnitude of electric field of TE mode for the complete optical path with two parallel SiN waveguides at a gap of 3.1 µm. Image shows that the field remains completely confined in the input waveguide.

EME analysis using MODE Solutions from Lumerical^®^ (Vancouver, Canada) was performed to study the effect of an air gap on optical signal transmission between two butt-coupled SiN channel waveguides with a core of 435 nm × 435 nm and with a top and bottom SiO_2_ cladding thickness of 3.4 µm for both the TE and TM modes. All of the optical simulations shown in this section were performed at a wavelength of 1550 nm. EME results show a transmission efficiency of over 99% for direct butt-coupling between these waveguides with an air gap of 50 nm or less, which is reduced to 33% when the gap is 3 µm. To reduce the expansion of the light beams in the gap and increase the coupling between the two waveguides, we introduced inverted tapers where the core width narrows down to 250 nm at both waveguide edges. The optimal length of the tapers was found to be 20 µm. The transmission efficiency is almost 100% with a 50 nm air gap and even on increasing the gap to 3 µm, the coupling efficiency dropped to 83% for the transverse electric (TE) mode and 74% for transverse magnetic (TM) mode in waveguides with inverted tapers. This result is shown in Figure 2a and demonstrates that a high coupling efficiency can be obtained even if fabrication imperfections limit the minimum size of the gap. Furthermore, the ability to reduce the gap to dimensions significantly smaller than the wavelength of light (which is typically around 1.3 µm or 1.5 µm in telecommunication applications) remove the need for an antireflection coating at the interface of the waveguides. When the gap size is larger than approximately half a wavelength, multiple beam interference phenomena can occur because of reflections at the waveguide interfaces, which explains the undulations that are visible in Figure 2a.

The dimensions of the central platform in an optical MEMS device as shown in Figure 1 are highly critical. The platform must accommodate at least three waveguides to operate as a 1 × 3 switch and four waveguides to operate as a 2 × 2 crossbar switch. Another important design consideration is the width of the gap closing interface between the platform and the substrate. The platform must be able to accommodate the number of waveguides envisioned with minimal optical cross-talk and optimal bending radius for low propagation losses [46]. Therefore, to have an estimate of the number of waveguides that can be implemented on the platform, we studied the cross-talk between two parallel SiN waveguides as function of the gap between them. 2.5D FDTD analysis were performed for 435 nm × 435 nm waveguides with a 3.4 µm thick top and bottom SiO_2_ cladding where the total length of the inner waveguide is 565.5 µm with a bending radius of 75 µm. It was found that for the TE mode the field remains confined in the input waveguide and does not couple to the adjacent outer waveguide when the gap between them is 3.5 µm or greater. The simulated propagation loss in the input waveguide of length 565.5 µm is only 0.01 dB for a 3.5 µm gap between adjacent waveguides. Results of the 2.5D FDTD cross-talk simulations are shown in Figure 2b. It can also be observed that the cross-talk for the TM mode is smaller than the TE mode and becomes negligible at a gap of 3.0 µm. 

**Figure 3:**
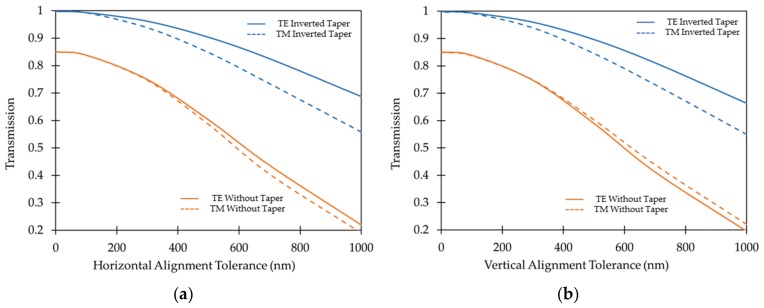
Optical simulation results for EME simulations showing the transmission efficiency of TE and TM modes between two butt-coupled SiN waveguides with and without inverted tapers as a function of: (**a**) horizontal and (**b**) vertical alignment tolerance.

The optical simulation results show that a rectangular platform of 150 µm by 520 µm is large enough to accommodate four separate SiN waveguides with a 75 µm bending radius. Also, the gap closing interface between the platform and the fixed section of the switch is 35 µm wide and can easily accommodate three separate 435 nm wide SiN waveguides with a 3.5 µm gap between them. These can be fabricated with inverted tapers having tip-width of 250 nm and 20 µm length in the coupling region at the edges for minimal optical loss. The transverse horizontal and vertical alignment tolerance between the butt-coupled waveguides with and without tapers were also analyzed as shown in Figure 3. The inverted tapered structures have a high alignment tolerance providing a transmission of more than 80% in case of the TE mode and of more than 70% in case of the TM mode even when one waveguide is displaced by 700 nm relative to the other. These transmission coefficients were obtained with an air-gap of 250 nm between the waveguides. Therefore, the proposed switch has high fabrication tolerances in comparison to typical silicon photonic devices implemented with SOI wafers that have a 220 nm thick device layer. 

### 2.3. MEMS Design Considerations

**Figure 4:**
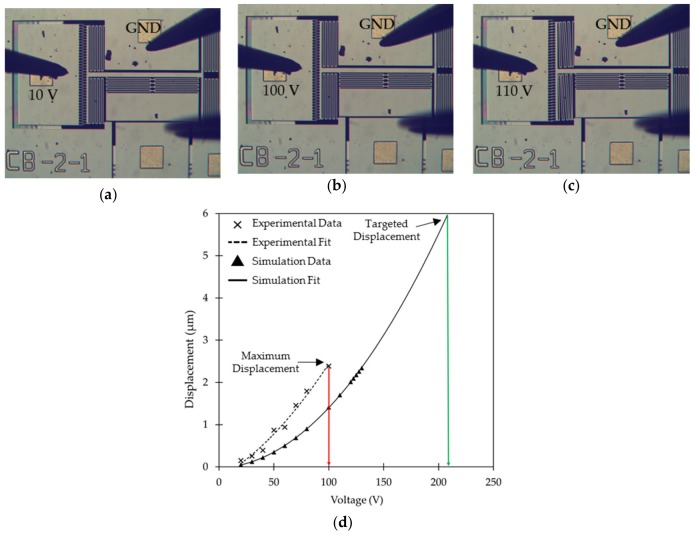
Microscopic micrographs of the translational MEMS platform with comb drive during actuation: (**a**) at 10 V; (**b**) maximum displacement at 100 V; (**c**) rotation at 110 V; (**d**) experimental and simulation based lateral switching displacement v/s actuation voltage curves for the translational MEMS design with comb drive.

**Figure 5:**
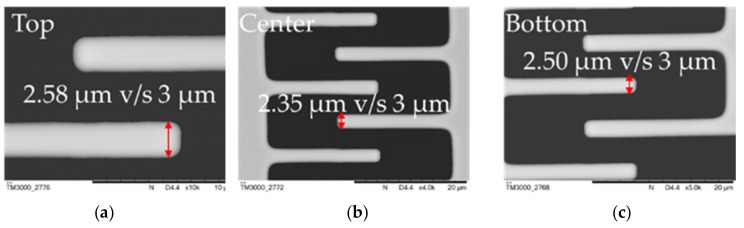
Fabricated v/s design dimensions through SEM micrograph analysis of the drive fingers for comb drive-based translational MEMS platform in various parts of the actuator: (**a**) top; (**b**) center; (**c**) bottom.

The initial MEMS actuator choice for the translational platform was to use of a unidirectional comb drive for lateral switching whereas the gap closing actuator was the same parallel plate actuator discussed above. This first MEMS design incorporated serpentine spring structures for both lateral and longitudinal motions. Since comb drives enable large controlled displacements, the MEMS was designed in ANSYS using static structural analysis to provide up to 6 µm of displacement at ~220 V. This design was fabricated by AEPONYX with an in-house microfabrication process for MEMS based on SOI technology. However, the fabricated devices showed rotational effects in the comb drive after a displacement of 2.39 µm at 100 V during testing. Figure 4 shows micrographs of a device during actuation tests along with the actuation curves for simulation and experimental results. The experimental measurements appear to follow a linear relationship in comparison to the simulation results because the displacements recorded during the experiment are limited to the beginning of the polynomial actuation curve where the slope is increasing slowly. Before we could observe the non-linear behaviour of the actuator, the comb drive-based actuator rotated inhibiting further actuation. SEM analysis of the MEMS device showed some fabrication discrepancies. Fabricated dimensions varied from 2.35 µm to 2.58 µm in the comb drive compared to the design dimensions of 3 µm. This varies the gap between the drive fingers in different regions of the comb. These observations are shown in Figure 5.

**Figure 6:**
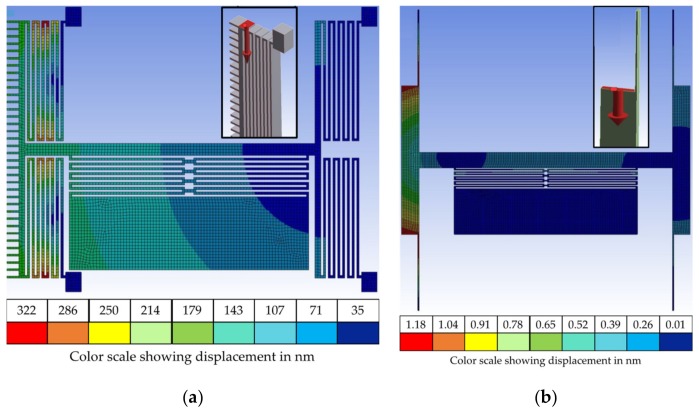
Total deformation heatmap when 10 µN of force is applied on the top left corner of the structure (force location shown in image insets): (**a**) comb drive and serpentine spring design; (**b**) parallel plate and single beam spring design.

The vertical in-plane stiffness of the main horizontal beam was further analyzed following the actuation results obtained. A static structural analysis of the structure was performed using ANSYS. A load force of 10 µN was simulated on the top left corner of the device model to verify the vertical stiffness of the main horizontal beam. These simulation results are shown in Figure 6a. The MEMS design has a low vertical stiffness as the 10 μN force applied led to a total maximum deformation of 322 nm. The fabrication discrepancies described earlier are assumed to make the electrostatic field generated by the fabricated comb drive slightly asymmetrical compared to the simulated model with an ideal comb drive. This is due to the varying gap between the fabricated fingers in different regions of the actuator. These fabrication geometry discrepancies combined with the low vertical stiffness of the system make the structure highly susceptible to the rotational effect observed. Designs for the lateral switching actuator and serpentine spring were modified to the final iteration shown in Figure 6b. Parallel plate actuators were chosen for lateral switching to simplify fabrication. The vertical stiffness was increased through a single beam spring for lateral actuation that is anchored on two ends unlike the previous serpentine spring design. A static structural simulation for a 10 µN force on the top left corner of the new design yielded only 1.18 nm of total maximum deformation, more than 300× reduction over the prior design shown in Figure 6a.

The completely parallel plate actuation-based design with a single beam spring was successful in eliminating any rotation due to the fabrication discrepancies caused by the complex comb drive structure, and to increase the vertical in-plane spring constant. In order to achieve the same targeted 6 µm of displacement as the comb drive, two lateral parallel plate actuators were designed on opposite sides of the platform.

## 3. Final Translational MEMS Platform 

The final iteration of the translational MEMS platform was designed on the basis of the optical design considerations and comb drive-based MEMS results discussed in the previous sections. Two parallel plate actuators were implemented on the opposite sides of the central rectangular platform for lateral displacement. Another parallel plate actuator was created on the bottom of the actuator for air gap closing. All actuators were designed as parallel-plate and operated under the pull-in effect [3]. In case of a parallel plate actuator with initial gap (*d*) and total overlap area (*A*) between the plates, the actuation voltage (*V_p_*) where the pull-in effect occurs is given by:

(1) $$V_{p}=\surd (\frac{8kd^{3}}{27\varepsilon A})$$

where *k* is the spring constant of the system in the direction of actuation and *ε* is permittivity of the dielectric medium. Also, the maximum controlled displacement (*X_p_*) for these actuators before pull-in is given by:

(2) $$X_{p}=\frac{d}{3}$$

**Figure 7:**
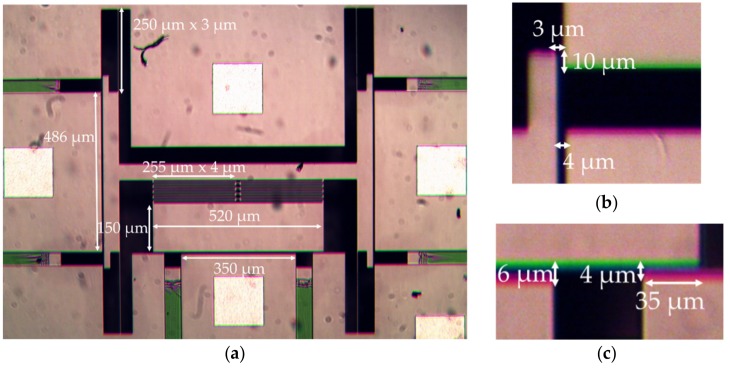
Micrographs of the final translational MEMS platform with critical design dimensions for: (**a**) platform, springs and actuators; (**b**) lateral switching actuator and stopper; (**c**) longitudinal gap closing actuator and stopper.

These lateral actuators were designed with an initial gap (*d*) of 4 µm making the maximum displacement (*X_p_*) before pull-in to be 1.3 µm. Similarly, for the longitudinal actuator with an initial gap (*d*) of 6 µm, the maximum displacement (*X_p_*) before pull-in is 2 µm. Since the pull-in effect enables a quick and large displacement, the two parallel plates in the actuator tend to snap together. In order to prevent shorting during pull-in, 10 µm long stoppers at a 3 µm gap (less than the actuator initial gap of 4 µm) were added at the two ends of both the lateral actuators. These stoppers also provide the necessary 3 µm of maximum displacement to translate the waveguides on the platform and form a 2 × 2 crossbar optical switch as discussed earlier. Similar 35 µm wide stoppers forming a 4 µm gap (less than the actuator initial gap of 6 µm) were built for the longitudinal actuator. These stoppers are larger than the lateral stoppers in order to accommodate multiple SiN waveguides. Images of the fabricated MEMS device along with the critical stopper and actuator dimensions are shown in Figure 7.

A static structural analysis for the device model in ANSYS was performed to estimate the lateral and longitudinal spring constants of the MEMS. The spring constant of the lateral actuators’ springs was found to be 15.37 N/m, whereas the spring constant of the platform’s serpentine spring is 1.81 N/m. The lower stiffness of the serpentine spring reduces the downward electrostatic force needed to close the gap. This helps limit the impact of gap closing actuation upon the lateral actuators and prevent any rotation of the platform. Since the stiffness of the serpentine spring is considerably lower, the gap closing actuator dimensions are different from the lateral actuators. The initial gap and length of the gap closing actuator was kept at 6 µm and 350 µm, respectively, whereas the initial gap and length of the lateral actuator was kept at 4 µm and 486 µm, respectively. These choices were made to enable the operation of all the actuators within a small voltage range. Theoretical calculations using the spring constant simulation results presented in this section predict a pull-in voltage of ~82 V and ~61 V for lateral switching and longitudinal gap closing, respectively. Modal analysis was also performed with the device model in ANSYS to obtain the resonance frequencies of the structure. The resonance frequency of the gap closing actuator was found to be 4.6 kHz whereas that of the switching actuator structure was 9.2 kHz. This limits the operational frequency for the switch at ~4.6 kHz. The MEMS cell as demonstrated can be used in both a 2 × 2 crossbar switch configuration as well as a 1 × 3 switch configuration.

## 4. Experimental Results

### 4.1. Microfabrication Results

**Figure 8:**
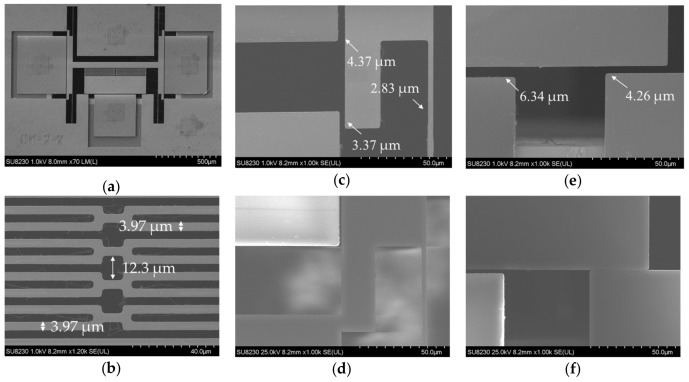
Detailed SEM micrographs with measurements of the fabricated translational MEMS platform: (**a**) final translational MEMS platform; (**b**) serpentine spring; (**c**) lateral actuator and spring’s fabricated dimensions; (**d**) lateral actuation during high power imaging; (**e**) gap closing actuator’s fabricated dimensions; (**f**) gap closing during high power imaging.

The MEMS devices were fabricated with a commercial process (PiezoMUMPs by MEMSCAP) [27]. The process uses SOI technology with a 10 µm device layer. SEM micrographs of the fabricated structures with measured critical dimensions are presented in Figure 8.

An analysis of the SEM micrographs showed that the fabricated dimensions were slightly different from the design dimensions. The lateral actuator gap increased from 4 µm to 4.37 µm and the stopper gap from 3 µm to 3.37 µm. The gap closing actuator gap increased from 6 µm to 6.34 µm and the stopper gap increased from 4 µm to 4.26 µm. These slight variations should increase the actuation voltage due to increased gap between the actuator plates. However, the spring beam dimensions were also smaller by a margin of ~0.17 µm for the lateral spring beams and by a margin of ~0.03 µm for the serpentine spring beams. This lowers the spring stiffness thereby negating the effect of the increase of the actuator gaps to some extent. A video showing both lateral switching and gap closing actuators in motion during SEM imaging is provided in the Supplementary Materials section. Actuation tests were performed to study the impact of these fabrication variations upon the actuation voltage. The test setup used, and the results obtained are presented in Section 4.2.

### 4.2. Actuation Test Results

**Figure 9:**
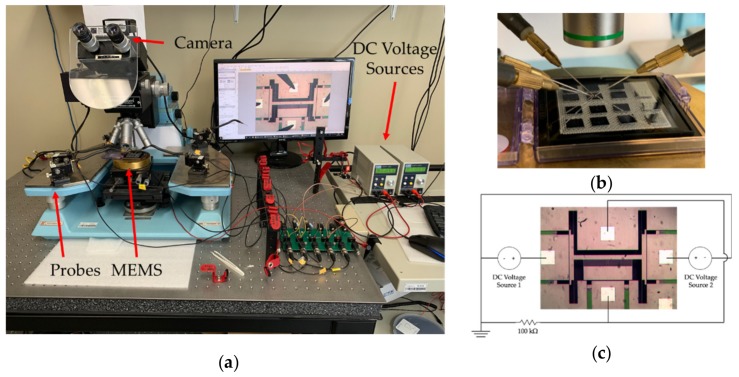
(**a**) Experimental actuation setup used; (**b**) zoomed in image of the probes on the MEMS device during tests; (**c**) schematic of the test circuit used for lateral actuation experiments.

Different fabricated devices were tested using a Wentworth probe station with a Bausch & Lomb microscopic system. Four DC probes were used during these tests. High voltage DC sources were used to provide the necessary voltage for actuation. A high resolution camera from Omax was used to image the devices during these tests. The actuator was grounded through a 100 kΩ resistor to prevent any device damage due to high current during actuation. Detailed image of the test setup used along with a schematic of the test circuit for the actuation experiments is given in Figure 9.

The lateral actuator on both the sides showed pull-in at an actuation voltage of 65 V. Since after pull-in the movable actuator plate snaps towards the fixed plate, the total displacement obtained should be equivalent to the gap between the actuator plates. However, 3 µm stoppers were included specifically in the design to prevent any shorting through contact between the two actuator plates. The fabricated dimensions for the devices discussed in Section 4.1 showed that a 3.37 µm stopper gap was fabricated instead. Our lateral actuator shows 3.37 µm of displacement for the central platform at just 65 V of pull-in voltage. Similarly, the results for the longitudinal gap closing actuator show pull-in at 50 V. The fabricated dimensions for the gap closing stoppers was 4.26 µm instead of 4 µm as per the design. The gap closing actuator provides a 4.26 µm displacement to the central platform at a pull-in voltage of just 50 V. No stiction issues or damage to the fabricated stoppers were observed after repeated actuation. The measured displacement for different actuation voltages of the lateral switching actuator follows a linear trend over the range of voltages used in the experimental characterization in comparison to the non-linear behavior of the gap-closing actuator before electrostatic pull-in. This can be explained by the difference in spring stiffness between the lateral switching and gap-closing actuators. The spring constant values for the single beam spring design of the lateral switching actuator in the simulation model was found to be 15.37 N/m, whereas that for the multi beam serpentine spring design of the gap-closing actuator is only 1.18 N/m. Therefore, the non-linear response of the gap-closing can be observed by applying a much smaller force or equivalently, a smaller actuation voltage. Also, the spring for the lateral switching actuator is similar to a clamped-clamped beam system which follows linear displacement as per small beam deflection theory up to a quarter of the beam thickness following which non-linear displacement can be observed for larger displacements [47]. Since the thickness of the SOI device layer used is 10 µm, the maximum displacement observed before pull-in is much lower than a quarter of the silicon beam thickness (i.e., 2.5 µm resulting in the linear behavior of the fabricated actuator). The simulation model for the lateral switching actuator shows an initial linear behavior which becomes non-linear with a larger displacement than seen in the measurements, which could be due to the higher stiffness of the spring by the actuator in the simulation model, due to the geometry variations resulting from the fabrication process, as seen in Figure 8. The experimental actuation voltage to reach pull-in was lower in comparison to the simulated model for both the lateral switching actuator and the gap-closing actuator. This can be explained by the difference in the fabricated and simulated silicon beam dimensions. As discussed earlier in Section 4.1, the width of the fabricated silicon beams in the spring structure was slightly less than in the simulation model. This reduces the stiffness of the spring leading to lower experimental actuation voltages for the simulated displacements compared to simulated actuation voltages. The experimental displacement before pull-in was also observed to be larger in comparison to the simulated model. This is because the fabricated gap between the parallel plates of the actuator was larger, as discussed earlier in Section 4.1.

**Figure 10:**
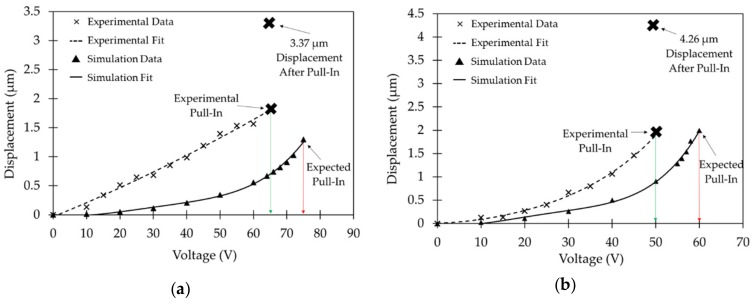
Experimental and simulation based displacement v/s actuation voltage results along with relevant pull-in voltages and maximum displacement obtained for (**a**) the lateral switching actuator; (**b**) the gap closing actuator.

**Figure 11:**
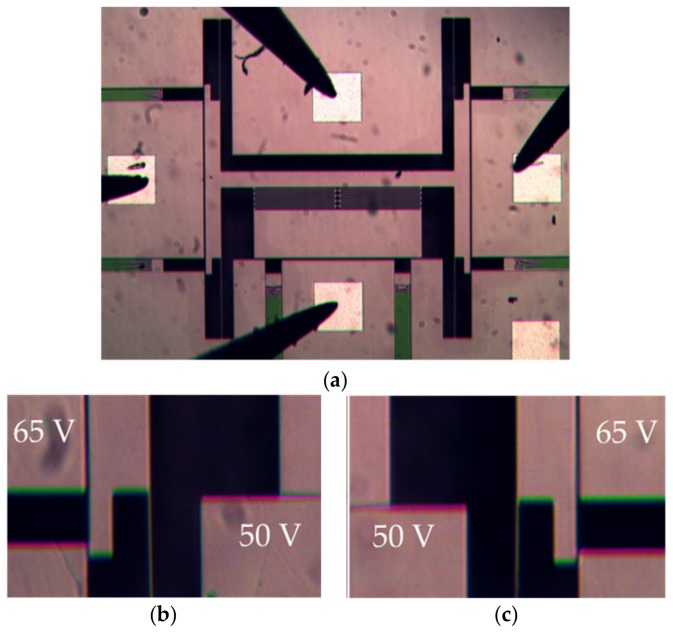
(**a**) Microscopic micrographs of the device at 0 V during actuation tests with probes on MEMS; (**b**) zoomed in image of the left switching actuator at 65 V and gap closing actuator at 50 V; (**c**) zoomed in image of the right switching actuator at 65 V and gap closing actuator at 50 V.

Simulation v/s experimental results are presented in Figure 10 and they show the pull-in voltage for both lateral switching and longitudinal gap closing actuators. Micrographs of the bi-directional lateral switching action at 65 V combined with vertical gap closing at 50 V along with the neutral position of the actuator are shown in Figure 11.

## 5. Discussion

Different translational MEMS actuator designs were implemented and tested. The comb drive-based actuator design showed a maximum displacement of 2.38 µm before a rotational effect occurred at 110 V precluding further motion. This maximum displacement of 2.38 µm achieved for the central platform could not provide the displacement of at least 6 µm needed for successful operation as a 2 × 2 crossbar switch. The MEMS design was improved through the incorporation of single beam spring for lateral switching actuation. Silicon beams anchored at two ends provide the necessary higher vertical stiffness and eliminated any rotational effect due to the serpentine spring structure with low vertical stiffness. Incorporation of simple parallel plate actuator design for lateral switching actuators eliminates any rotational effect caused due to fabrication discrepancies in the comb drive geometry. Two parallel plate actuators on the opposite side of the central platform provide the necessary discrete displacement of at least 6 µm (i.e., 3 µm on each side) after pull-in for lateral switching. Parallel plate actuator designed for air gap closing motion of the central platform provides zero gap between platform and the substrate upon actuation. Soft spring design for the central platform using serpentine spring system also ensures zero impact of the air gap closing motion upon the lateral switching motion of the platform.

The state-of-the-art planar optical switch developed in [41] demonstrates low loss switches which rely upon polarization sensitive vertical adiabatic coupling between polysilicon ridge waveguides. Although polarization insensitive switches based upon polysilicon waveguides have also been realized, these involve a complex 20 masks fabrication process with 3 waveguide layers [48]. State-of-the-art 2 × 2 MEMS switches with zero gap butt-coupling between suspended and fixed waveguides has also been demonstrated in the past through the incorporation of soft polymer waveguides over the MEMS actuator and had a low switching speed of < 0.5 ms. This approach involves a complex bonding process between the polymer waveguides and MEMS structures [39]. Also, the actuator springs need to be precompressed into latching position using probes under a microscope to provide the zero gap coupling between waveguides and optical fibers.

The translational platform presented in this work is designed to be integrated with polarization insensitive square SiN waveguides in a single optics layer with SiO_2_ cladding for less stringent packaging requirements. The actuator springs designed do not require any complex assembly procedure before switching operation for zero gap closing either. This is due to the independent spring design for lateral switching and air gap closing motions which provides bi-axial motion to the central platform necessary for its operation in 2 × 2 crossbar switch configuration. Recently, a rotational MEMS platform demonstrated crossbar switching capability at 118 V with the ability to reduce the air gap between fixed and movable waveguides down to 250 nm at an actuation voltage of 113 V [42]. The translational platform presented in this work operates at a much lower voltage of 65 V for 2 × 2 crossbar switching and 50 V for air gap closing. The rectangular platform design also provides the unique possibility to integrate SiN based optical filters on the platform itself in 1 × 3 switch configuration.

Although the design choice of parallel plate-based actuation for lateral switching makes the alignment of the optical waveguide mask with the MEMS mask during microfabrication critical, previously SiN waveguides have been successfully integrated with high precision [42]. Also, optical simulations of the alignment tolerances showed more than 80% efficiency for 700 nm of misalignment and more than 96% efficiency for less than 300 nm of misalignment with a 250 nm gap between the waveguides. Stepper tools for lithography can be used to precisely align the MEMS layer with the waveguides during microfabrication process. Optical simulations show that the gap closing motion of the platform can provide over 99% transmission efficiency for butt-coupling waveguides. Waveguides with inverted tapers can provide more than 83% efficiency even when there is a separation of 3 μm between them. The design is capable of minimizing the optical losses due to the air gap. The effect of surface roughness of the fabricated devices upon the minimal gap achievable should not cause significant optical losses either.

## 6. Conclusions

In this work, a translational MEMS platform was presented for planar optical switching applications. The lateral switching actuator designed for this translational MEMS device operates at an actuation of voltage of 65 V while closing the air gap completely at just 50 V. The ability to integrate up to four SiN waveguides with minimal crosstalk on the large 150 µm by 520 µm platform provides 2 × 2 crossbar switching capability. A 2 × 2 crossbar switch can be realized with just one core switch cell in a smaller device footprint of 1 mm by 1 mm when compared to [42]. This switch also operates at a much lower voltage when compared to the rotational MEMS platform designed for planar crossbar switching. It can also be used to demonstrate a wavelength channel selection system through integration with SiN based optical filters on the central platform for Reconfigurable Optical Add–Drop Multiplexer (ROADM) applications [49,50]. In the future, we aim to integrate SiN waveguides and optical filters with the fabrication process demonstrated previously [42]. Spring stiffness and actuator dimensions will be further optimized so that both lateral and gap closing actuators operate at the same voltage. Actuator stopper dimensions will also be optimized for minimal stiction and high reliability. 

## Supplementary Materials

The following are available online at https://www.mdpi.com/2072-666X/10/7/435/s1, Video S1: Translational MEMS platform in motion.
